# Relationship between allergic rhinitis and diamine oxidase activity: A preliminary report 

**DOI:** 10.5414/ALS400537

**Published:** 2021-06-18

**Authors:** Miguel Mayo-Yáñez, Andrea Díaz-Díaz, Juan C. Vázquez-Barro, Jesús Herranz González-Botas, Angélica Figueroa, Carlos S. Martín-Martín

**Affiliations:** 1 Otorhinolaryngology, Head and Neck Surgery Department, University Hospital Complex of A Coruña (CHUAC), A Coruña, Galicia,; 2 Clinical Research in Medicine, International Center for Doctorate and Advanced Studies (CIEDUS), University of Santiago de Compostela (USC), Santiago de Compostela, Galicia,; 3 Epithelial Plasticity and Metastasis Group, Biomedical Research Institute of A Coruña (INIBIC), University Hospital Complex of A Coruña (CHUAC), University of A Coruña (UDC), A Coruña, Galicia,; 4 School of Educational Sciences, University of A Coruña (UDC), A Coruña, Galicia,; 5 School of Medicine and Odontology, University of Santiago de Compostela (USC), Santiago de Compostela, Galicia, Spain, and; 6 Otorhinolaryngology, Head and Neck Surgery Department, University Hospital Complex of Santiago de Compostela (CHUS), Santiago de Compostela, Galicia, Spain

**Keywords:** allergic rhinitis, diamine oxidase, histamine intolerance, nasal obstruction, histamine

## Abstract

Aim: To analyze the diamine oxidase (DAO), the main catabolic enzyme of histamine, degradation activity and its relation with symptoms of persistent allergic rhinitis. Methods: In this descriptive and analytical observational study, we collected DAO activity levels and the nasal peak inspiratory flow. Results: Enzymatic activity deficit in 108 patients was 46.3% (95% CI, 0.44 – 0.63), 33.33% in mild and 47.92% in moderate/severe rhinitis (p = 0.376). The nasal peak inspiratory flow in patients with a deficit in DAO activity was 76.30 ± 28.40 L/min compared to 93.62 ± 37.50 L/min in patients with normal enzymatic activity (p = 0.010). Conclusions: It seems that the lower the catabolic activity of DAO, the lower the nasal peak inspiratory flow observed. Although DAO activity levels could be a severity biomarker in allergic rhinitis, a cause-effect association cannot be concluded. The enzyme could be another actor in the pathophysiology of allergic rhinitis.

## Introduction 

Allergic rhinitis (AR) is an inflammation of the mucous lining clinically defined by nasal symptoms induced by an immunologically mediated reaction after the exposure of the nasal mucous membranes to an offending allergen. These symptoms include rhinorrhea, nasal obstruction, nasal itching, sneezing, and postnasal drip [1]. 

Histamine is the main mediator, producing nasal airway exudation, itching, and obstruction in subjects with AR through an immune reaction [2, 3, 4]. Mast cells and vascular endothelial cells synthesize and store it from the decarboxylation of the amino acid L-histidine, and its catabolism is regulated mainly by diamine oxidase (DAO). In mammals, this enzyme is expressed in specific tissues, especially the gastrointestinal tract, placenta, and kidney [2, 5, 6]. Under normal circumstances, DAO forms an enzymatic barrier in cells of the intestinal epithelium, which sufficiently protects from resorption of histamine from ingested food into the blood stream [2, 5, 6, 7]. 

A failure in the function of this enzyme results in an imbalance of the ingested histamine and the capacity for its degradation [2, 5, 6]. Some clinical studies have correlated DAO deficiency with specific pathologies, mainly gastrointestinal, dermatological diseases or migraine [6, 8, 9, 10, 11, 12, 13, 14, 15], depending on the expression of histamine receptors (H1 – H4) in tissues [16]. However, there is little information available about DAO function in patients diagnosed with AR and its correlation with symptoms [17, 18, 19]. The aim of this study is to analyze the DAO degradation activity in patients with persistent AR. 

## Methods 

### Study design and data collection 

This was a descriptive and analytical observational cross-sectional study in adult patients diagnosed with persistent AR, recruited from the outpatient departments of the Otorhinolaryngology, Head and Neck Surgery Department of a tertiary hospital. Selection of the volunteer participants was performed through a structured interview conducted by one Otorhinolaryngologist and one Allergologist. This research involved human participants and was approved by the Hospital’s Ethics Committee (Code: 2016/106). Written informed consent was obtained from all individual participants included in the study. 

Inclusion criteria were to have a confirmed diagnosis of persistent AR according to the Allergic Rhinitis and its Impact on Asthma (ARIA) guidelines criteria [1], to not present important nasoseptal deformities or polyps objectivated by physical examination (anterior rhinoscopy and flexible fibronasoendoscopy, and to have not been treated for the past 2 weeks with DAO enzyme or any medication that could not be suspended and causes acquired decrease (antihistamines, systemic glucocorticosteroids, aminophylline, cefuroxime, clavulanic acid, metoclopramide, verapamil, etc.) or increase (heparin) in DAO activity [2, 5, 7]. Additionally, patients with a 2-week intake history of foods containing histamine such as fermented foods, beverages, processed meat, and seafood, or patients with any other medical disorders (hepatic, gastrointestinal, or renal disease, deficiency of vitamin B6, vitamin C, copper, or zinc, etc.) or pregnant were excluded from this study [2, 5, 7]. 

Sociodemographic variables, DAO enzyme activity, the health-related quality of life ESPRINT-15 questionnaire [20], the degree of severity according to the ARIA guidelines (mild and moderate/severe) [1], and the nasal peak inspiratory flow (NPIF) were collected [21, 22]. 

### ESPRINT-15 questionnaire 

The ESPRINT-15 (Cuestionario ESPañol de Calidad de Vida en RINiTis) is a specific quality of life questionnaire for allergic rhinitis, validated in the Spanish population and with reference values that facilitate its proper interpretation. It has 15 items in 4 dimensions (symptoms, daily activities, sleep, and psychological well-being), with scores from 0 to 6. Lower scores indicate better quality of life [23]. Reference values in persistent AR were (men: mild = 0.5, moderate/severe = 2.6; women: mild = 0.8, moderate/severe = 2.7) [20, 22, 24]. 

### Nasal peak inspiratory flow 

NPIF is a simple and rapid technique that is carried out using a plastic tube (20 cm long, 3 – 4 cm in diameter, and calibrated at 30 – 370 L/min) to which a face mask is attached (GM Instruments Ltd., Irvine, UK). From an expiratory maneuver to residual volume, a forced inspiration is made while the lips are sealed. Three measurements that must not vary by more than 10% are taken, and the best one is chosen. Normal values for adult males (Caucasian) were 143 ± 48.6 L/min and for adult females (Caucasian) 121.9 ± 36 L/min [21, 22]. 

### DAO activity analysis 

Blood samples were collected from all subjects by venipuncture in an EDTA tube after an 8-hour fasting period. Samples were analyzed with ELISA to determine DAO enzyme activity in accordance with the manufacturer’s instructions (D-HIT, Sciotec Diagnostic Technologies GmbH, Tulln an der Donau, Austria). This method was previously used for the same purpose by Mušič et al.[25] Values above 80 HDU/mL (histamine degrading Unit/mL) were considered normal, while values below 80 HDU/mL were considered DAO deficient. One HDU corresponds to the DAO activity that degrades 1 pmol/mL of histamine. 

### Statistical analysis 

Statistical analysis was performed with the statistical package R 3.6.1 (The R Foundation for Statistical Computing, Vienna, Austria). Statistical tests were two-tailed with a 95% confidence interval (CI). A minimum sample size of 97 randomly selected subjects was calculated to estimate, with a precision ± 8% units, a population DAO deficiency percentage considered to be ~ 20%, based on previous literature [6, 11, 17]. Normality was evaluated by the Kolmogorov-Smirnov test and variances using the Levene test. Quantitative variables were expressed as mean ± standard deviation (SD) and median. The comparison of means between groups was performed using the Student’s t, Mann-Whitney, ANOVA, or Kruskal-Wallis test as appropriate. Qualitative variables were expressed as frequency and percentage. The differences between groups were evaluated by the χ^2^-test, Fisher’s exact test or its variants as appropriate. In cases where non-normality was significant, non-parametric methods were applied (Wilcoxon test and Kruskal-Wallis test). 

## Results 

### Descriptive analysis 

A total of 108 Caucasian patients with persistent AR were recruited, 36 (33.33%) men and 72 (66.67%) women. The mean age was 32.91 ± 12.8 years, 34.15 ± 11.79 years for men and 32.28 ± 13.31 years for women (p =0.323), respectively. 64.15% of the population were non-smokers, and the remaining percentage was divided between smokers (n = 18; 16.98%) and ex-smokers (n = 20; 18.87%), with no gender differences (p = 0.070). According to the ARIA severity of the symptoms, 12 (11.11%) patients had mild rhinitis (4 men and 8 women), and 96 (88.89%) had moderate/severe rhinitis (32 men and 64 women), with no gender differences. 

### DAO enzyme activity 

The mean blood determination of the DAO enzyme activity was 91.20 ± 40.81 HDU/mL, being 96.66 ± 37.88 HDU/mL in men and 88.47 ± 42.19 HDU/mL in women (p = 0.101). The prevalence of DAO activity deficit was 46.3% (95%CI, 0.44 – 0.63, p < 0.000). The mean activity was 118.29 ± 37.49 HDU/mL in the group with normal function compared to 59.77 ± 11.19 HDU/mL in the group with deficit (p < 0.000). 

Depending on the rhinitis severity, the mild group had a prevalence of DAO deficit of 33.33% compared to the moderate/severe group, with a 47.92% (p = 0.376). The mean DAO activity in patients with mild rhinitis was 110.22 ± 52.07 HDU/mL compared to 88.83 ± 38.87 HDU/mL of patients with moderate/severe rhinitis (p = 0.119). Considering the smoking habit, no differences were found in DAO activity levels (p = 0.518) or the prevalence of DAO activity deficit (p = 0.089). The rest of the activity values are described in Table 1. 

### Nasal peak inspiratory flow 

The mean NPIF was 85.60 ± 34.55 L/min, being 100.83 ± 36.42 L/min for men and 77.98 ± 31.13 L/ min for women (p = 0.001). In the mild AR group, NPIF was 107 ± 32.85 L/min, and in the moderate/severe group it, was 82.91 ± 33.97 L/min (p = 0.016). Considering the smoking habit, the NPIF was 89.71 ± 37.69 L/min for non-smokers, 89 ± 30.71 L/min for ex-smokers, and 68.88 ± 15.77 L/min for smokers (p = 0.034). 

**Figure 1.**
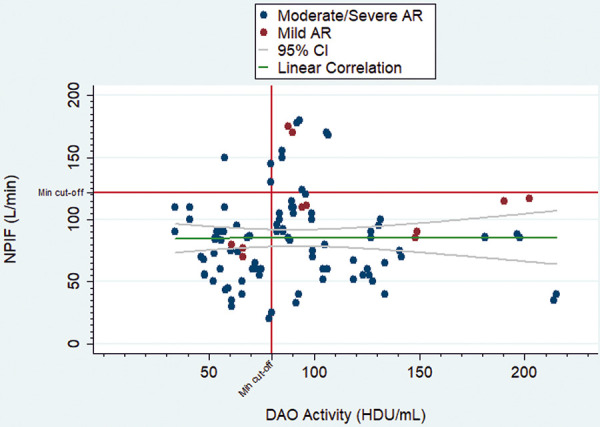
Correlation between diamine oxidase activity levels and nasal peak inspiratory flow according to allergic rhinitis severity group. The minimum cut-off points for both measurements are indicated.

The NPIF in patients with a deficit in DAO activity was 76.30 ± 28.40 L/min compared to 93.62 ± 37.50 L/min in patients with normal enzymatic activity (p = 0.010). No linear correlation was found between DAO activity levels and NPIF in the whole sample (ρ = 0.04; p = 0.676) (Figure 1). In the group with mild AR, the NPIF was 81.25 ± 7.5 L/min for DAO deficiency and 120 ± 33.16 L/min for normal enzymatic activity (p = 0.031). In the group with moderate/severe AR severity, it was 75.86 ± 29.53 L/min for the deficit activity and 89.4 ± 36.71 L/min for normal activity (p = 0.057). The results are shown in Table 1. 

### Symptomatology 

The typical symptoms of rhinitis and histamine intolerance were evaluated according to DAO activity (Table 2). A patient with DAO activity deficit has a higher risk of suffering from migraine at least twice a month (OR = 2.89; p = 0.041), gastrointestinal discomfort (OR = 2.72; p = 0.012), and constipation/diarrhea without apparent cause (OR = 2.62; p = 0.016). 

### ESPRINT-15 

The ESPRINT-15 questionnaire reached a mean score of 2.7 ± 1.21, being 2.5 ± 1.30 in men and 2.76 ± 1.15 in women (p = 0.319). Depending on the severity, patients with mild rhinitis had a score of 0.9 ± 0.3, and patients with moderate/severe rhinitis had a score of 2.8 ± 1 (p < 0.000). According to DAO activity, no significant differences were found (p = 0.815), with a score of 2.7 ± 1 in the activity deficit group and 2.6 ± 1.3 in the normal activity group. No linear correlation was found between the ESPRINT-15 score and the DAO activity levels (rho = –0.06; p = 0.543). 

## Discussion 

AR is among the most common diseases worldwide and generally persists throughout life. It has been estimated that the prevalence of referred AR is ~ 2 – 25% in children and 1% to more than 40% in adults [1]. The mechanisms of the allergic reaction are being better understood over time, although histamine remains one of the main factors of the allergic reaction. A genetic or acquired error in the enzymatic catabolic function of DAO causes an imbalance of accumulated histamine and the capacity for its metabolization, and this is called histamine intolerance [2, 5, 6]. This relationship between the enzyme and the amount of extracellular histamine could play a role in the pathophysiology of the allergic diseases and therefore, in persistent AR. Although some studies measured DAO intracellularly or in serum,[19, 26] this study did not measure the quantity but the activity of the enzyme. Therefore, this study was the first to analyze the possible relationship between an imbalance in the metabolism of histamine due to a DAO activity deficit and the symptoms of persistent AR. 

There are few reports that have studied the relationship between AR and DAO deficit [17, 18, 19]. Some pointed to the possible use of DAO levels as an allergic biomarker in this type of patient [19], while others pointed out that there could be subgroups of AR patients with a different disease course due to single nucleotide polymorphisms (SNPs) that would cause DAO enzyme malfunction and an increased histamine accumulation even in situations where the amount of DAO is within normal parameters [6, 17, 18]. Specifically, the most relevant SNPs affecting DAO enzyme functionality in Caucasian individuals, like our population, are rs10156191, rs1049742, rs2268999, and especially rs1049793 [27]. This study is the first to measure the DAO enzymatic activity and to perform an objective measurement of the nasal flow using the NPIF in persistent AR. 

The results show that about half of the patients with AR suffer from a deficit of DAO activity. These data are consistent with the high prevalence found in other pathologies [6, 13, 16]. Despite this, the results should be viewed with caution, requiring their validation in future control group studies. Likewise, the presence of other symptoms typically associated with histamine intolerance was evaluated, showing that the presentation of gastrointestinal symptoms and migraine is more frequent in patients with a deficiency of enzyme activity. These results are consistent with those observed in previous studies evaluating this type of pathology, in which a decrease in DAO activity was found in 80 – 90% of patients [6, 13, 16]. On the other hand, the presence of dermatological symptoms was not associated with a deficit in DAO activity, although the presence of both entities simultaneously does not seem to be so clear, being found only in small subgroups in the literature [11, 12, 28]. The low specificity and complex variability of symptoms undoubtedly contribute to the current difficulty in creating associations between symptoms and DAO deficit, as well as achieving consensus on the diagnostic criteria for histamine intolerance [6, 29, 30]. 

Patients with moderate/severe rhinitis had lower enzyme activity compared to patients with mild rhinitis. It seems logical that if a greater accumulation of histamine occurs, the severity of AR will be greater. This finding could be useful in future studies, making it possible to use the DAO activity levels as a biomarker to differentiate between severity groups in AR [19]. There is a higher degree of nasal obstruction measured objectively by NPIF in patients with DAO activity deficiency, regardless of the severity group of rhinitis. The lower the catabolic activity of the DAO enzyme, the lower the NPIF observed, but without a linear association between parameters. With these results, and as it was suggested in previous studies, the clinical course of AR might be altered in patients with impaired histamine metabolism [17]. There are many causes of malfunction or decrease in DAO activity [5, 11, 31, 32, 33] For example, carriers of DAO C2029G mutated allele tend to develop more severe symptoms of rhinitis and other histamine intolerance-related symptoms [17, 18, 34]. Therefore, in future studies this fact must be corroborated. 

Despite this association, no significant differences or correlations were found in the quality of life measured with the ESPRINT-15 questionnaire between the groups with normal DAO activity and deficient DAO activity. This is in contrast to recently described findings in which linear correlations were obtained between AR severity and blood DAO levels [19]. Therefore, it is convenient to differentiate between the level of DAO in blood (previous studies) and its activity (current study). Our results indicate that variations in DAO function do not affect the quality of life of patients with AR despite the fact that they may be associated with a worsening of the NPIF. 

Among the limitations of this study, it is important to highlight the impossibility of analyzing the real prevalence of DAO activity deficit, since patients were recruited solely from an outpatient department and the data collected could thus not be extrapolated to the general AR population. Subjects presenting only mild and intermittent symptoms may not recognize their condition as treatable, which could influence patient motivation to attend the consultation or accept being referred from primary care services. This may also apply to subjects presenting symptoms all year round and who may therefore consider their symptoms simply as a fact of life rather than a disease requiring treatment [35, 36]. This could be one of the reasons for the distribution of mild and moderate/severe AR found. Other limitations would be the cross-sectional design without a control group, the lack of other determinations (IgE, histamine, allergens), the lack of follow-up, or the lack of repeated DAO activity levels in the same patient [37, 38]. The strengths are the measurement of the NPIF and to relate it for the first time with DAO activity and histamine intolerance in AR, but even the analysis of DAO activity has been called into question as a diagnostic method for histamine intolerance [10, 25, 29, 30, 39]. This study opens up a range of possibilities, but the degree of the relationship found might not be enough to define the real link between DAO activity and AR severity. 

On one hand, a cause-effect association between the severity of AR and the DAO activity cannot be concluded because AR is an entity in which many mediators are involved and possibly DAO is just one more actor in its pathophysiology. However, DAO activity could be considered in the future as a biomarker to differentiate between severity groups. On the other hand, there is some controversy about which measure is the best to assess DAO, whether its levels or its activity [19, 25, 29, 39, 40, 41, 42]. This work demonstrates that the same conclusions are not applicable for both parameters. Future studies in which the two parameters are evaluated together are necessary to discern the meaning and importance of each. 

Finally, in recent years there is a tendency to relate the DAO activity deficit, and the consequent histamine intolerance, with a wide spectrum of signs and symptoms that can be improved with a low-histamine diet or enzyme supplementation therapy [14, 15, 31, 40, 43, 44, 45, 46]. Therefore, if the association between DAO activity deficiency and AR is confirmed, it could be considered as a therapeutic target for AR. 

## Funding 

None. 

## Conflict of interest 

There was no conflict of interest. 

**Table 1.** Diamine oxidase enzyme activity values and nasal peak inspiratory flow according to groups.

		HDU/mL			L/min		
	N (%)	Mean ± SD	Median	p-value	Mean ± SD	Median	p-value
Population	108	91.20 ± 40.81	84.14	–	85.60 ± 34.55	85	
DAO activity deficit	50 (46.3)	59.77 ± 11.19	59	**< 0.000**	76.30 ± 28.40	75	**0.010**
Normal DAO activity	58 (53.7)	118.29 ± 37.49	105.27		93.62 ± 37.50	90	
Men	36 (33.33)	96.66 ± 37.88	92.09	0.101	100.83 ± 36.42	95	**0.001**
Women	72 (66.67)	88.47 ± 42.19	77.09		77.98 ± 31.13	77.5	
Severity groups							
Mild	12 (11.11)	110.22 ± 52.07	92.09	0.118	107 ± 32.85	100	**0.016**
Moderate/Severe	96 (88.89)	88.83 ± 38.87	83		82.91 ± 33.97	80	
Mild							
Normal DAO activity	8 (66.67)	133.57 ± 48.86	121.18	**0.008**	120 ± 33.17	112.5	**0.031**
DAO deficit	4 (33.33)	63.52 ± 3.28	63.52		81.25 ± 7.5	80	
Moderate/severe							
Normal DAO activity	46 (47.92)	115.85 ± 35.35	105.27	**< 0.000**	89.4 ± 36.71	85	0.057
DAO deficit	50 (52.08)	59.44 ± 11.59	57.68		75.86 ± 29.53	72.5	

DAO = diamine oxidase; HDU = histamine degrading unit; SD = standard deviation. **Bold** = statistically significant.

**Table 2.** Typical nasal symptoms and histamine intolerance-related symptoms.

	Normal activity N (%)	DAO deficit N (%)	OR	p-value
Nasal symptoms				
Anosmia	38 (65.52)	26 (56.52)	0.68	0.349
Nasal Obstruction	50 (86.21)	44 (95.65)	3.52	0.105
Sneezing	54 (93.1)	44 (95.65)	0.15	0.580
Itching	44 (75.85)	46 (100)	**0**	**< 0.000**
Posterior rhinorrhea	36 (62.07)	26 (56.52)	0.79	0.567
Anterior rhinorrhea	48 (82.76)	38 (82.61)	0.98	0.984
Hit symptoms				
Migraine > 2/month	6 (10.34)	12 (25)	**2.89**	**0.041**
Constipation/diarrhea	18 (31.03)	26 (54.17)	**2.62**	**0.016**
Gastrointestinal discomfort	22 (37.93)	30 (62.5)	**2.72**	**0.012**
Musculoskeletal pain	46 (79.31)	34 (70.83)	0.63	0.313
Skin problems	12 (20.69)	10 (20)	0.96	0.432
Generalized fatigue	30 (51.72)	20 (41.67)	0.67	0.302
Fibromyalgia	2 (3.45)	2 (4.17)	1.22	1
Asthma	22 (37.93)	20 (41.67)	1.17	0.695

OR = odds ratio; HIT = histamine intolerance. **Bold** = statistically significant.
